# Entanglement-Based CV-QKD with Information Reconciliation over Entanglement-Assisted Link

**DOI:** 10.3390/e26040305

**Published:** 2024-03-29

**Authors:** Ivan B. Djordjevic, Vijay Nafria

**Affiliations:** Department of Electrical and Computer Engineering, University of Arizona, 1230 E. Speedway Blvd., Tucson, AZ 85721, USA; nafriavijay@arizona.edu

**Keywords:** entanglement, QKD, continuous variable, entanglement assisted communication, information reconciliation

## Abstract

An entanglement-based continuous variable (CV) QKD scheme is proposed, performing information reconciliation over an entanglement-assisted link. The same entanglement generation source is used in both raw key transmission and information reconciliation. The entanglement generation source employs only low-cost devices operated in the C-band. The proposed CV-QKD scheme with information reconciliation over an entanglement-assisted link significantly outperforms the corresponding CV-QKD scheme with information reconciliation over an authenticated public channel. It also outperforms the CV-QKD scheme in which a classical free-space optical communication link is used to perform information reconciliation. An experimental demonstration over the free-space optical testbed established at the University of Arizona campus indicates that the proposed CV-QKD can operate in strong turbulence regimes. To improve the secret key rate performance further, adaptive optics is used.

## 1. Introduction

With the help of entanglement [1,2,3,4,5,6,7,8,9], we can: beat the capacities of classical channels [1,2,3,4,5,10,11,12], achieve better sensitivity than classical sensors [1,4], and provide quantum-mechanics-based security [1,3,6]. The security of quantum key distribution (QKD) is guaranteed by quantum-information-processing theorems, such as the no-cloning theorem and theorem on the indistinguishability of arbitrary quantum states, rather than computation complexity [3,6,7,8,9]. Different photon degrees of freedom can be utilized in QKD, including polarization, time, frequency, phase, and orbital angular momentum. Among the different QKD protocols, discrete variable (DV)-QKD and continuous variable (CV)-QKD are very popular ones. In DV-QKD schemes, a single-photon detector (SPD) is applied, while in CV-QKD, we rely on the uncertainty principle. With CV-QKD, we can achieve higher secret key rates (SKRs) compared to corresponding DV-QKD schemes, thanks to its compatibility with state-of-the-art telecom optical communications [3]. For a comprehensive introduction to CV-QKD systems, interested readers are referred to [13] (see also [3]). The research on CV-QKD is gaining momentum, judging by the increasing number of papers published in the last two decades [14,15,16,17,18,19,20,21,22,23,24,25,26,27,28,29,30]. Generally speaking, CV-QKD schemes can be classified as Gaussian-modulation- or discrete-modulation-based. Further, they can be either entanglement-based or coherent-states-based. Typically, information reconciliation is performed over the authenticated public channel, to which Eve has access [31].

In this paper, we are concerned with the entanglement-based free-space optical (FSO) CV-QKD scheme, in which reverse information reconciliation is performed over an entanglement-assisted communication link. Entanglement-assisted communication is based on the principles described in ref. [10,11,12]. To reduce the system complexity and cost, the same entanglement generation source is used for both raw key transmission and information reconciliation. Instead of using a high-cost 780 nm pump laser to implement the entangled source, we develop the entanglement generation source using only low-cost telecom devices operated in the C-band. To experimentally evaluate the proposed CV-QKD system, we develop a free-space optical testbed at the University of Arizona campus with a propagation path length of 1.5 km. We experimentally demonstrate that, in strong turbulence regimes, the proposed CV-QKD scheme with information reconciliation over an entanglement-assisted link significantly outperforms the corresponding conventional scheme performing information reconciliation over the authenticated classical channel. To improve the secret key rate performance, adaptive optics [32,33,34,35] is used.

The paper is organized as follows. In Section 2, the proposed entanglement-based CV-QKD scheme with information reconciliation over the entanglement-assisted link is described. In Section 3, we describe the terrestrial FSO CV-QKD testbed that we developed at the University of Arizona campus. The experimental results are provided in Section 3. Some important concluding remarks are given in Section 4.

## 2. Proposed Entanglement-Based CV-QKD with Information Reconciliation over Entanglement-Assisted Links

**Figure 1:**
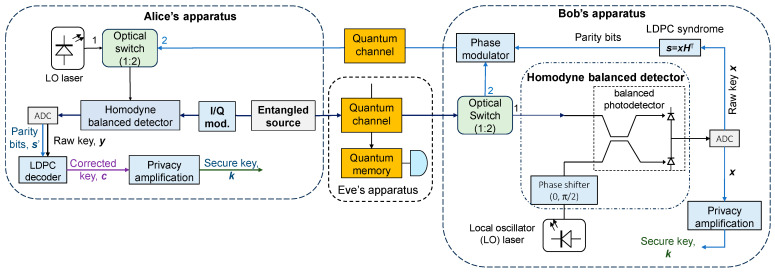
Illustrating the proposed entanglement-based CV-QKD scheme with information reconciliation over an entanglement-assisted link.

The proposed entanglement-based CV-QKD scheme with information reconciliation over an entanglement-assisted link is provided in Figure 1. The entanglement generation source, based on parametric down-conversion (PDC), is placed on the Alice side. The PDC entangled source generates two-mode squeezed vacuum (TMSV) states, which can be represented on the basis of the number (Fock) states as follows:

(1) $${|\psi \rangle }_{A,B}={\left(N_{s}+1\right)}^{-1/2}\sum_{n=0}^{\infty }{\left(\frac{N_{s}}{N_{s}+1}\right)}^{n/2}{|n\rangle }_{A}{|n\rangle }_{B},$$

where $N_{s}=\langle {\hat{a}}_{A}^{†}{\hat{a}}_{A}\rangle =\langle {\hat{a}}_{B}^{†}{\hat{a}}_{B}\rangle$ is the mean photon number corresponding to either Alice (A) or Bob (B) qubits. Alice and Bob photon creation (annihilation) operators are described by ${\hat{a}}_{A}^{†}\;({\hat{a}}_{A})$ and ${\hat{a}}_{B}^{†}\;({\hat{a}}_{B}).$ The phase-sensitive cross-correlation (PSCC) coefficient $\langle {\hat{a}}_{A}{\hat{a}}_{B}\rangle =\sqrt{N_{s}(N_{s}+1)}$ is related to the Alice–Bob photon pair entanglement.

The Wigner covariance matrix of the pure maximally entangled zero-mean Gaussian TMSV state is given by [1]:

(2) $$\Sigma_{A,B}=\left[\begin{array}{cc} (2N_{s}+1)\;\text{1} & 2\sqrt{N_{s}(N_{s}+1)}\;Z\\ 2\sqrt{N_{s}(N_{s}+1)}\;Z & (2N_{s}+1)\;\text{1} \end{array}\right],$$

where **1** denotes the identity matrix and ***Z*** is the Pauli *Z*-matrix. Evidently, in a regime with *N_s_* << 1, the phase-sensitive cross-correlation coefficient is $\langle {\hat{a}}_{A}{\hat{a}}_{B}\rangle \approx \sqrt{N_{s}}$, and in comparison with the classical limit *N_s_*, we obtain that $\sqrt{N_{s}}\gg N_{s}.$

By using the electro-optical I/Q modulator, Alice randomly selects a point in the signal space (phase space). With the optical switch in position 1, Alice performs homodyne balanced detection on her qubit photons by mixing them with local oscillator (LO) photons using a directional coupler-based balanced detector. By setting the phase-shift after the LO oscillator to either 0 or π/2, Alice selects measuring either the in-phase or quadrature component. Bob’s qubit photons at the output of the entangled source are transmitted over the quantum channel. With the optical switch in position 1, Bob randomly measures either the in-phase on quadrature component with the help of his homodyne balanced detector. By selecting the instances where Alice and Bob measured the same component, after corresponding analog-to-digital converters (ADCs), Bob and Alice obtain the raw keys ***x*** and ***y***, respectively.

In the conventional scheme, Alice and Bob will further perform the information reconciliation over the public channels, to which Eve has access to. In our proposed solution, we perform reverse information reconciliation over an entanglement-assisted system, as shown in Figure 1, by employing the same entangled source used for raw key transmission. With both optical switches in position 2, Bob performs LDPC encoding to obtain the parity bits ***s***. With the help of a phase modulator, the parity bits are imposed on Bob’s qubit photons and transmitted over the same quantum channel in the opposite direction. Alice then performs homodyne balanced detection on the photons received from Bob by using her qubit’s photons as the reference photons. Given that Bob and Alice’s photons are entangled, the information reconciliation is performed over the entanglement-assisted system. Following the balanced homodyne detection, Alice performs LDPC decoding to obtain the correct key identical to Bob’s one. Finally, Alice and Bob perform privacy amplification to remove any correlation with Eve and, thus, obtain a secure key.

Based on refs. [1,3,8,9], we can calculate the normalized secret key rate (SKR) as follows:

(3) $$r=\beta I\left(A;B\right)-\chi \left(B;E\right),$$

where *β* is the reconciliation efficiency, *I*(*A*;*B*) is the mutual information between Alice and Bob, and *χ*(*B*;*E*) is the Holevo trans-information between Bob and Eve, denoted as *χ*(*B*;*E*). The mutual information between Alice and Bob is identical for both individual and collective attacks and is given by [1,3,31]:

(4) $$I\left(A;B\right)=\frac{1}{2}\log_{2}\left(\frac{v+\chi_{\mathrm{total}}}{1+\chi_{\mathrm{total}}}\right),$$

where *v* is the variance of the source, while the variance of the total noise, denoted as $\chi_{\mathrm{total}}$, is obtained by the summing up the variance of the channel noise $\chi_{\mathrm{line}}$ and the homodyne detection noise $\chi_{\mathrm{homodyne}}$, which can be expressed by referring to the *channel input* by:

(5) $$\chi_{\mathrm{total}}=\chi_{\mathrm{line}}+\frac{\chi_{\mathrm{homodyne}}}{T};\;\chi_{\mathrm{line}}=\;\frac{1-T}{T}+\varepsilon ;\;\chi_{\mathrm{homodyne}}=\frac{1-\eta +v_{el}}{\eta },$$

where *T* is the transmittance of the channel, *v_el_* is the photodiodes’ electrical noise, *η* is the detector efficiency, and *ε* is the excess noise that accounts for the modulation imperfections, the phase noise, and the relative intensity noise (RIN) of the LO reference signal, etc. The Holevo trans-information between Bob and Eve is determined by [1,3,8,9]:

(6) $$\chi \left(B;E\right)=g\left(\frac{\lambda_{1}-1}{2}\right)+g\left(\frac{\lambda_{2}-1}{2}\right)-g\left(\frac{\lambda_{3}-1}{2}\right)-g\left(\frac{\lambda_{4}-1}{2}\right),$$

where $g\left(x\right)=\left(x+1\right)\log_{2}\left(x+1\right)-x\log_{2}x$ and *λ_i_* are the symplectic eigenvalues of corresponding covariance matrices determined by:

(7) $$\lambda_{1,2}=\sqrt{\frac{1}{2}\left(A\pm \sqrt{A^{2}-4B}\right)},\;\;\lambda_{3,4}=\sqrt{\frac{1}{2}\left(C\pm \sqrt{C^{2}-4D}\right)},$$

with the corresponding parameters *A*, *B*, *C*, and *D* being defined by:

(8) $$\begin{array}{l} A=v^{2}\left(1-2T\right)+2T+T^{2}{\left(v+\chi_{\mathrm{line}}\right)}^{2},\;\;B=T^{2}{\left(1+v\chi_{\mathrm{line}}\right)}^{2},\\ C=\frac{A\chi_{\mathrm{homodyne}}+v\sqrt{B}+T\left(v+\chi_{\mathrm{line}}\right)}{T\left(v+\chi_{\mathrm{total}}\right)},\;D=\sqrt{B}\frac{v+\chi_{\mathrm{homodyne}}\sqrt{B}}{T\left(v+\chi_{\mathrm{total}}\right)}. \end{array}$$

The reconciliation efficiency in the proposed CV-QKD scheme is determined by:

(9) $$\beta =\frac{R}{I\left(B;A\right)},$$

where *R* is the code rate of the LDPC code used in the reverse information reconciliation (RIR), while *I*(*B*;*A*) is the mutual information between the Bob and Alice channel used in the RIR, which is different from the one used in Equation (4), as discrete modulation is used in RIR, while Gaussian modulation in raw key transmission.

## 3. Description of Terrestrial Free-Space Optical (FSO) Testbed to Study the Proposed Entanglement-Based CV-QKD Scheme

**Figure 2:**
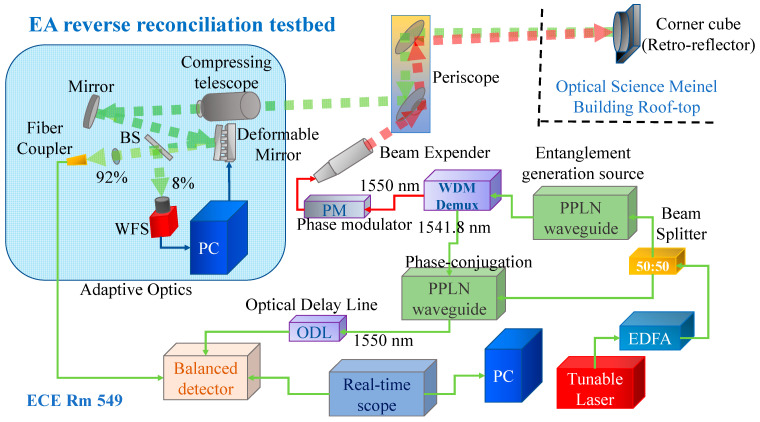
The reverse information reconciliation entanglement-assisted testbed developed at the University of Arizona campus.

To evaluate the SKR performance of the proposed entanglement-based CV-QKD scheme, we developed an FSO testbed at the University of Arizona campus. In Figure 2, we describe the reverse reconciliation entanglement-assisted (EA) communication testbed, which is composed of the following stages: (1) entanglement generation source, (2) WDM demultiplexer-based stage to separate the signal and idler photons, (3) modulation stage, (4) transmission stage, (5) beam collection and compression stage, (6) stage to delay the idler photons, (7) homodyne balanced detection stage, and (8) bit-error rate (BER) computing stage. The experimental setup is located in ECE Room 549 of the ECE building at the University of Arizona, where the Quantum Communication (QuCom) Lab is located. The entanglement generation source employs only low-cost telecom devices operated in the C-band. A tunable laser set to 1545.9 nm is used as the pump laser, whose output is amplified by the high-power EDFA and split into two parts using a 50:50 beam splitter. The top beam splitter output is used as the input to a type-0 periodically poled lithium niobate (PPLN) waveguide. In this PPLN waveguide, entangled photon pairs are generated by processes of secondary harmonic generation (SHG), followed by spontaneous parametric down-conversion (SPDC). The entanglement pair we selected are 1550 nm as signal photons and 1541.8 nm as idler photons. The signal photons are modulated using a phase modulator, which is modulated by the RF signal from an arbitrary waveform generator (AWG) set to 10 Gb/s, in which the LDPC-encoded BPSK information sequence is recorded. We then transmit the phase-modulated signal photons out of the Lab towards a retro reflector placed around 750 m away on the roof top of the Optical Sciences Meinel building. By using the mirror in the middle of the link, we can establish connection when there is no line-of-sight between Alice and Bob. The reflected beam at the ECE 549 lab window, after a round trip of ~1.5 km, is collected by a periscope and compressing telescope. On the other hand, we perform optical phase-conjugation on the idler photons by mixing them with a 1545.9 nm amplified pump signal and passing them through the bottom PPLN waveguide, thus performing the difference frequency generation. The output of the bottom (phase-conjugation) PPLN waveguide is passed through a WDM demultiplexer, we select a 1550 nm output, and the phase-conjugated photons are propagated over 1 km of SMF, which serves as the optical delay line (ODL). After collecting the signal photons by a compressing telescope, we pass them over the optical bench with an adaptive optics setup and finally couple them in an optical fiber. The signal photons and idler photons are fed into a balanced homodyne detector and the RF output of the balanced detector is recorded by a real-time oscilloscope running at a sampling rate of 100 GSa/s. The recorded waveforms are then processed by the PC to perform uncoded BER calculation, LDPC decoding, post-forward error correction (FEC) BER calculation, and reverse information reconciliation efficiency calculation. Further, to provide improvements in BER in the information reconciliation stage and SKR, an adaptive optics (AO) setup is used, where the beam after the compressing telescope goes through an AO setup, which is composed of a deformable mirror (DM) and a wavefront sensor (WFS) operated in a servo loop.

**Figure 3:**
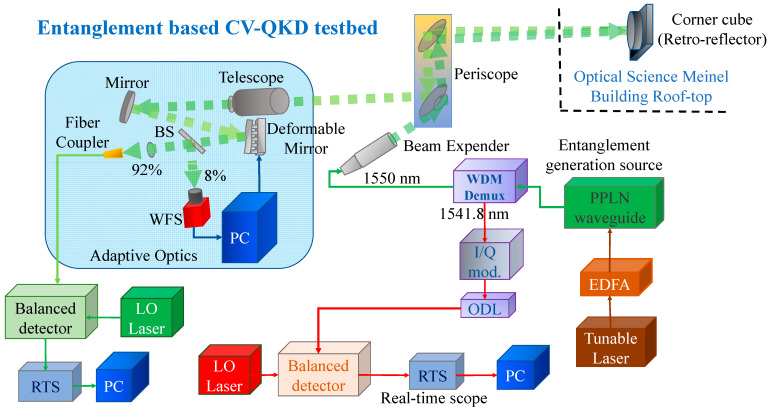
The entanglement-based CV-QKD free-space optical testbed.

The corresponding entanglement-based FSO testbed for the raw key transmission study is provided in Figure 3.

Similarly to Figure 2, the same entangled source is used for raw key transmission. Bob’s qubit photons at 1550 nm are transmitted over the 1.5 km FSO link. Alice’s 1541.8 nm qubit photons are modulated by the I/Q modulator. The ODL matches the FSO propagation time of Bob’s photons. Alice mixes her qubit photons on an optical hybrid before the balanced detector (not shown in Figure) with LO photons and randomly measures either the in-phase or quadrature component with the homodyne balanced detector. Bob mixes his photons received by the compressing telescope on an optical hybrid and selects to measure either the in-phase or quadrature component of the received signal. Bob announces his selections of the measured component, but keeps the results of the measurement private. Bob and Alice keep the instances when they measured the same component as the raw keys ***x*** and ***y***, respectively.

Bob’s encodes his raw key by the LDPC code, selected based on the FSO channel conditions, imposes such an encoded BPSK sequence with the phase modulator, and transmits it over the entanglement-assisted scheme shown in Figure 2. The rest of the protocol is the same as that described in the previous section. Corresponding experimental results are provided in the next section.

## 4. Experimental Results

**Figure 4:**
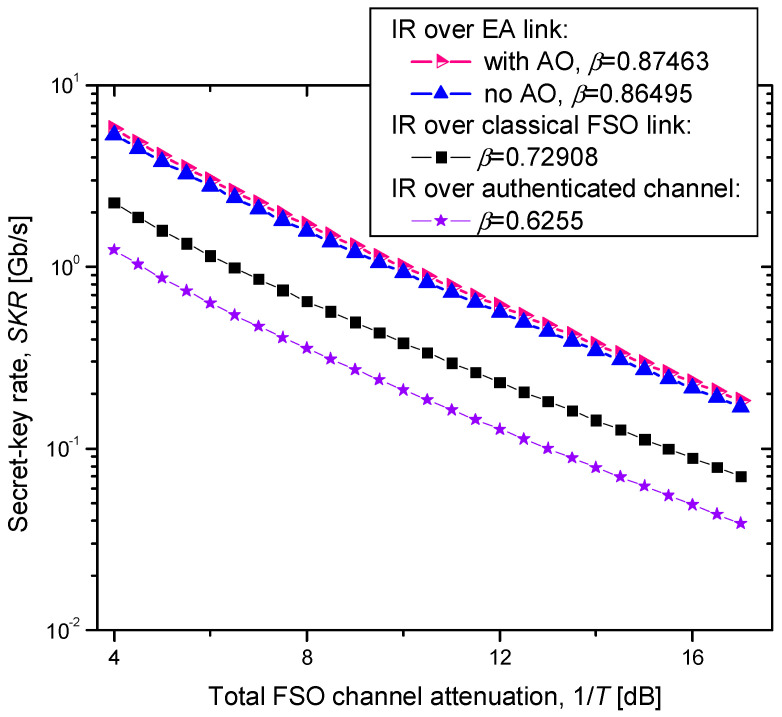
SKR vs. total channel attenuation experienced over the FSO link for different information reconciliation (IR) schemes. The raw key signaling rate was 10 Gb/s.

**Figure 5:**
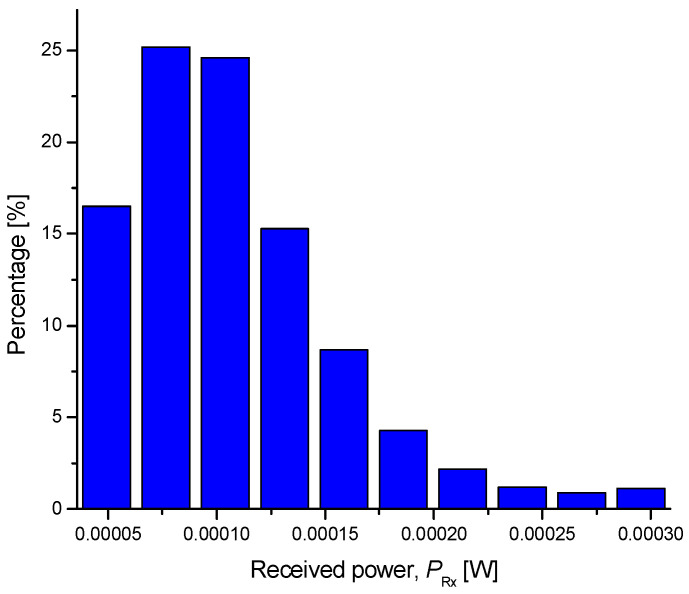
The histogram of received power during experiments.

The secret key rate results are summarized in Figure 4 for a raw key rate of 10 Gb/s. The calculations are based on Equations (3)–(9), by employing the calibration method described in ref. [7,8]. The histogram of the received power, provided in Figure 5, has a Rayleigh distribution, which indicates that the experimental demonstrations were conducted in a strong turbulence regime [32,33]. In Figure 4, we show the SKR results for different values of channel attenuations experienced during raw key transmissions. The system parameters were found to be *η* = 0.8, *V_el_* = 0.0321, and *ε* = 0.011. We compare the SKRs for different reverse information reconciliation (IR) schemes: the proposed EA-based IR scheme with and without adaptive optics, the IR based on the classical communication scheme operated over the FSO link, and the IR over the authenticated public channel. Clearly, the proposed EA-based IR scheme outperformed all the other schemes for the range of the total FSO channel attenuation found for the duration of the experiments. The achievable reconciliation efficiencies are provided in the figure. For a total channel loss of 10 dB, the SKR of the proposed scheme was 0.92 Gb/s, while the SKR of IR over the classical FSO link was 0.39 Gb/s. The SKR of the corresponding scheme with IR over the authenticated public channel was only 0.21 Gb/s. Therefore, the CV-QKD with the IR over the EA link significantly outperformed the corresponding conventional scheme with IR over the authenticated channel. In strong turbulence regimes, the adaptive optics provide some improvement in the SKR. Even though the improvement in the SKR for the AO (in strong turbulence regime) was small, it is still relevant in the information reconciliation stage in order to improve the BER and reduce the FEC frame loss.

## 5. Concluding Remarks

An entanglement-based CV-QKD scheme was proposed, performing information reconciliation over an entanglement-assisted communication link. In the proposed scheme, the same entanglement generation source, developed by employing spontaneous parametric down-conversion, was used for dual purposes: raw key transmission and information reconciliation. The developed entanglement generation source employed low-cost telecom devices operated in the C-band. To evaluate the proposed CV-QKD scheme, a free-space optical testbed was developed at the University of Arizona campus with a propagation path of length of 1.5 km. Experimental verification showed that, in a strong turbulence regime, the proposed CV-QKD scheme with information reconciliation over an entanglement-assisted link was capable of significantly outperforming the corresponding CV-QKD scheme performing the information reconciliation over an authenticated public channel. The proposed scheme was also shown to outperform the CV-QKD scheme in which a classical FSO communication link is used to perform the information reconciliation. It was found that, in a strong turbulence regime, adaptive optics does not provide significant improvements in the secret key rates.

## Data Availability

The data presented in this study are available on request from the corresponding author.
